# Supplementary figures and images for: Repetitive Compressive Loading Downregulates Mitochondria Function and Upregulates the Cartilage Matrix Degrading Enzyme MMP-13 Through the Coactivation of NAD-Dependent Sirtuin 1 and Runx2 in Osteoarthritic Chondrocytes
Source: Int J Mol Sci. 2025 May 22;26(11):4967. doi: 10.3390/ijms26114967 (PMC12155324; doi:10.3390/ijms26114967)

## Supplementary data

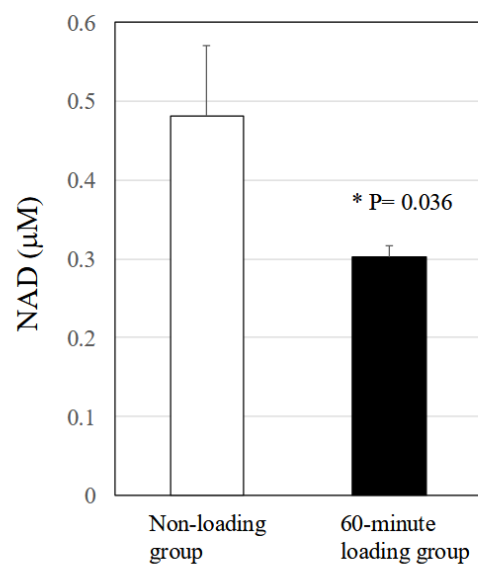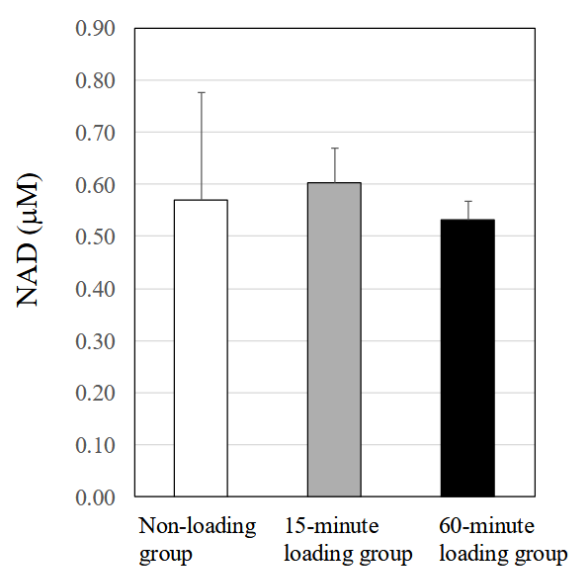

Supplement: Supplementary file 1 [file ijms-26-04967-s001.zip › ijms-3603289-supplementary.pdf]
